# How the Brain Analyzes Object Shape to Support Grasping Behavior

**DOI:** 10.1371/journal.pbio.1002071

**Published:** 2015-02-17

**Authors:** Janelle Weaver

**Affiliations:** Freelance Science Writer, Carbondale, Colorado, United States of America

**Fig. 1:**
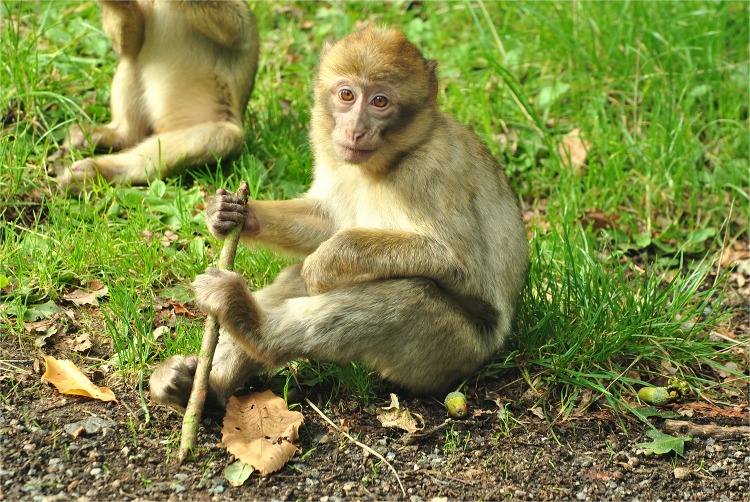
Reaching out and grasping involve the coordination of several brain regions. Here, the authors use electrical microstimulation while scanning the brains of macaque monkeys to elucidate the organization of neuronal networks that support grasping behaviour. Image credit: John5199, Flickr.

Reaching out and grasping an object is second nature to us, but this simple task requires various brain regions to analyze visual features, such as the 3-D shape of the object, and use that information to plan and execute actions (Fig. 1). Past studies have shown that neurons in a brain region called the anterior intraparietal area (AIP) respond selectively to 3-D shape and are active during object grasping. But there has been a lack of research on the causal effects of AIP neuronal activity on other brain regions involved in visual analysis and motor planning, limiting knowledge about the organization of neuronal networks that support grasping behavior.

In a study published this week in *PLOS Biology*, Peter Janssen, of KU Leuven, and his collaborators combined two unique approaches to shed light on the function of AIP and its fine-scale connections with other parts of the brain. Using electrical microstimulation and functional magnetic resonance imaging (EM-fMRI) in macaque monkeys, the researchers discovered that the posterior subsector of AIP is a critical site of convergence of the dorsal and ventral visual processing streams, which are involved in analyzing the location and identity of objects, respectively. The findings provide new insights into the organization of the network of brain regions involved in analyzing 3-D shape to support behavior.

The researchers first identified 3-D shape-selective sites in AIP by recording neural activity in monkeys presented with pictures of convex and concave surfaces. Both the anterior and posterior parts of the AIP, which in some cases were separated by no more than 3 mm, contained a high proportion of neurons that were selective for 3-D shape. Then they used electrodes to electrically stimulate those sites while recording brain activity with fMRI. Stimulation of the anterior AIP led to an increase in neural activity in a network of somatosensory and motor areas implicated in reaching and grasping. By contrast, stimulation of the posterior AIP increased neural activity in brain regions involved in object processing.

These findings demonstrate that neighboring AIP neurons with similar response properties are connected with markedly different networks of brain regions in parietal, temporal, and frontal cortices. This distinction between the posterior and anterior portions of AIP could not be readily detected in anatomical studies, highlighting the crucial advantage of the EM-fMRI approach over anatomical tracer studies for identifying the connections of specific clusters of neurons with subsectors of other brain regions. Unlike tracer experiments, EM-fMRI studies do not require animals to be sacrificed, so animals are spared and the same animals can then be used to further the same line of investigation. According to the authors, this study is the first to apply EM-fMRI to the macaque AIP, and it provides the first causal evidence relating the properties of individual neurons to their in vivo neural connections in posterior parietal cortex.

Taken together, the patterns of neuronal connections strongly suggest that 3-D shape information is transmitted from posterior AIP to anterior AIP and subsequently to the motor system—a collection of brain regions involved in producing body movements. Before contact with an object, AIP regions may access information about object identity through connections with the ventral stream to help with selecting the appropriate grasp.

Moreover, the results help to explain several anatomical and physiological observations. For example, neurons in the posterior AIP tend to be more responsive to visual features, whereas neurons in the anterior AIP tend to be more active during grasping behavior. This visual-to-motor gradient in the AIP can now be linked to connections between the posterior AIP and the ventral stream as well as connections between the anterior AIP and somatosensory and motor areas.

In summary, the results clearly demonstrate that the posterior AIP is a pivotal brain region where the dorsal and ventral visual streams interact during object analysis. More broadly, the study shows that charting the connections of functionally defined patches of neurons provides novel insights into the widespread neuronal networks that support behavior.
